# IMAAAGINE: a webserver for searching hypothetical 3D amino acid side chain arrangements in the Protein Data Bank

**DOI:** 10.1093/nar/gkt431

**Published:** 2013-05-28

**Authors:** Nurul Nadzirin, Peter Willett, Peter J. Artymiuk, Mohd Firdaus-Raih

**Affiliations:** ^1^School of Biosciences and Biotechnology, Faculty of Science and Technology, Universiti Kebangsaan Malaysia, 43600 UKM Bangi, Malaysia, ^2^Information School, University of Sheffield, Western Bank, Sheffield S10 2TN, UK and ^3^Department of Molecular Biology and Biotechnology, Krebs Institute, University of Sheffield, Western Bank, Sheffield S10 2TN, UK

## Abstract

We describe a server that allows the interrogation of the Protein Data Bank for hypothetical 3D side chain patterns that are not limited to known patterns from existing 3D structures. A minimal side chain description allows a variety of side chain orientations to exist within the pattern, and generic side chain types such as acid, base and hydroxyl-containing can be additionally deployed in the search query. Moreover, only a subset of distances between the side chains need be specified. We illustrate these capabilities in case studies involving arginine stacks, serine-acid group arrangements and multiple catalytic triad-like configurations. The IMAAAGINE server can be accessed at http://mfrlab.org/grafss/imaaagine/.

## INTRODUCTION

A number of tools now exist to search the Protein Data Bank (PDB) (1) for similar patterns to existing protein structures both at the fold level e.g. (2) and at the level of clusters of amino acids (3–9). These methods use query patterns that are derived from atomic structures solved by crystallography and nuclear magnetic resonance spectroscopy. However, in contrast to these searching methods, in this article, we describe a server that is designed to allow the interrogation of the PDB for hypothetical side chain patterns that may or may not occur in reality. This method is therefore not limited to existing coordinate sets and does not require pre-constructed atomic models. Crucially, a reduced structural representation is built into the program to facilitate more fluid open-ended searching. The program IMAAAGINE presents an easy-to-use interface that permits the specification of queries consisting of between three and eight residues, with the necessity of defining only a subset of the possible distances between them. The Ullmann subgraph isomorphism algorithm (10) is then used to search the PDB for matching structures. The use of the minimal side chain description provides a simple but effective mechanism for specifying generic queries and thus for enhancing the recall of a search. Generic side chains such as acid, base and so forth can also be deployed in the search query. As illustrated in the case studies section, these capabilities enable the user to use his or her imagination to create novel patterns and then to discover whether such side chain arrangements exist within the database of known structures or whether an ‘unusual’ side chain arrangement is actually unprecedented.

## PROGRAMS AND METHODS

The underlying concept behind the search methodology for IMAAAGINE is based on that used in SPRITE and ASSAM (8), but with major differences designed to allow much more freedom in the search process. Briefly, in all three programs, the protein structure is represented as a graph with the nodes representing individual amino acid side chains and the edges representing the inter-node geometric relationships (in terms of distances in 3D space). In ASSAM and SPRITE, each node consisted of two pseudo-atoms (representing the Start and End of each side chain, with a Midpoint position also available) that were used to generate a vector, and each such vector corresponded to one of the nodes in a graph (8). Up to five distances between pairs of vectors representing each pair of side chains therefore allowed definition of the relative angular rotations between the side chains. We also showed that the ASSAM and SPRITE representation, which was based on side chain positions rather than main chain positions, had distinct advantages in detecting side chain–side chain motifs over main chain-based representations that did not (8).

**Figure 1.**
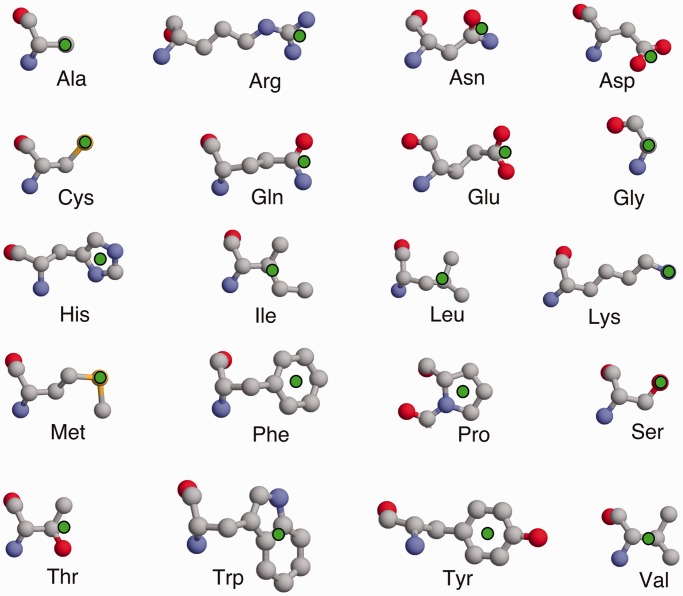
The side chain representation used in IMAAAGINE. The 20 amino acid types are shown with the position of the Key pseudo-atom position indicated by a green filled circle.

In IMAAAGINE, however, a single Key pseudo-atom is used to represent the functional part of each side chain. The position of this pseudo-atom is designed to emphasize the most important functional part of the side chain, and a diagram of the Key positions is shown in Figure 1. The Key position is intended to focus on the most characteristic part of that particular kind of amino acid side chain. Thus, for the basic residues Lys, Arg and His, we chose pseudo-atoms centred on their regions of positive charge, namely, the ends of the Lys and Arg side chains and at the centre of the imidazole moiety of a His. For acid groups, we chose to place the pseudo-atom at the centre of negative charge on the end of the carboxyl group and for amides at the equivalent position on the amide. For serine, we chose the OG atom where the hydroxyl is situated, for cysteine the SG atom and for threonine the average of OG1 and CG2. These choices enable us also to define mutually compatible generic basic, acidic, amide, charged and hydrophilic groups, which can be equivalenced to one another within the search tolerances. For hydrophobic residues, we chose pseudo-atoms in the centres of their hydrophobic part, once again allowing generic hydrophobic or aromatic residue types. This means that only the distances between single points on each side chain are specified, thereby allowing significant degree of rotational freedom between side chains. Moreover, so long as every side chain has at least one specified distance to at least one other, it is not necessary to define all or even most of the inter-residue distances, as the distances that are not defined become ‘wildcard’ distances that are able to adopt any value.

This ability to allow the majority of inter-residue distances to be undefined permits great freedom to the search procedure as illustrated in the case study examples. ASSAM (8) on the other hand had a more intricate representation, which reflected the angular as well as distance orientation of residues, and all distances had to be defined. To facilitate the use of partially defined distance queries, we used the Ullmann subgraph isomorphism algorithm (10) in IMAAAGINE rather than the Bron and Kerbosch maximal common subgraph algorithm (11) used in the ASSAM webserver (8).

As will be illustrated in the ‘Case Studies’ section, IMAAAGINE allows great flexibility in searching, which is enhanced by the use of a search tolerance on those distances that are specified. In addition, we believe that the interface is sufficiently intuitive and clear to be useable by non-structural biologists with an interest in structural information.

## IMAAAGINE: INPUT INTERFACE

**Figure 2.**
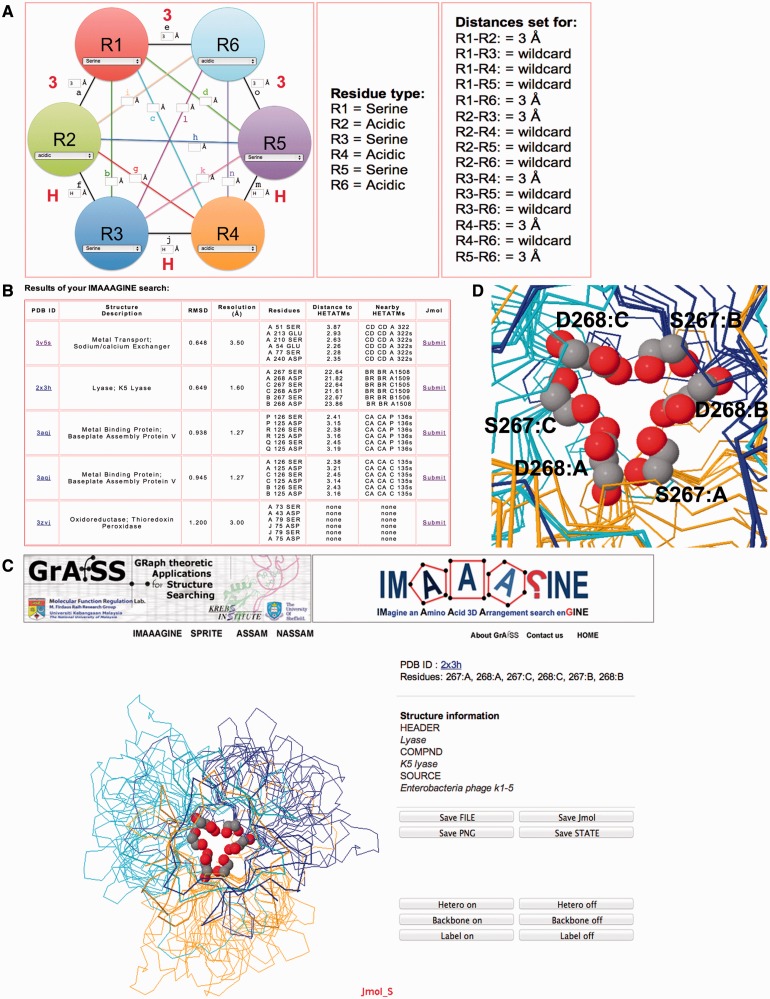
Examples of an IMAAAGINE query design interface and output. (**A**) The main input interface (left panel) for providing the type of amino acid residue and distances that define the arrangement between the residues, whereas the character H and numeral 3 in red are magnifications of the input in the boxes that can also be tracked on the centre and right panels. (**B**) An example of a search carried out for the query specified in (A). (**C**) An example of the web browser embedded visualization of IMAAAGINE hits using Jmol. (**D**) A magnification for one of the hits for the search in (A) detailing the occurrence of an SDSDSD motif in the structure of a phage lyase trimer (PDBID: 2X3H).

**Figure 3.**
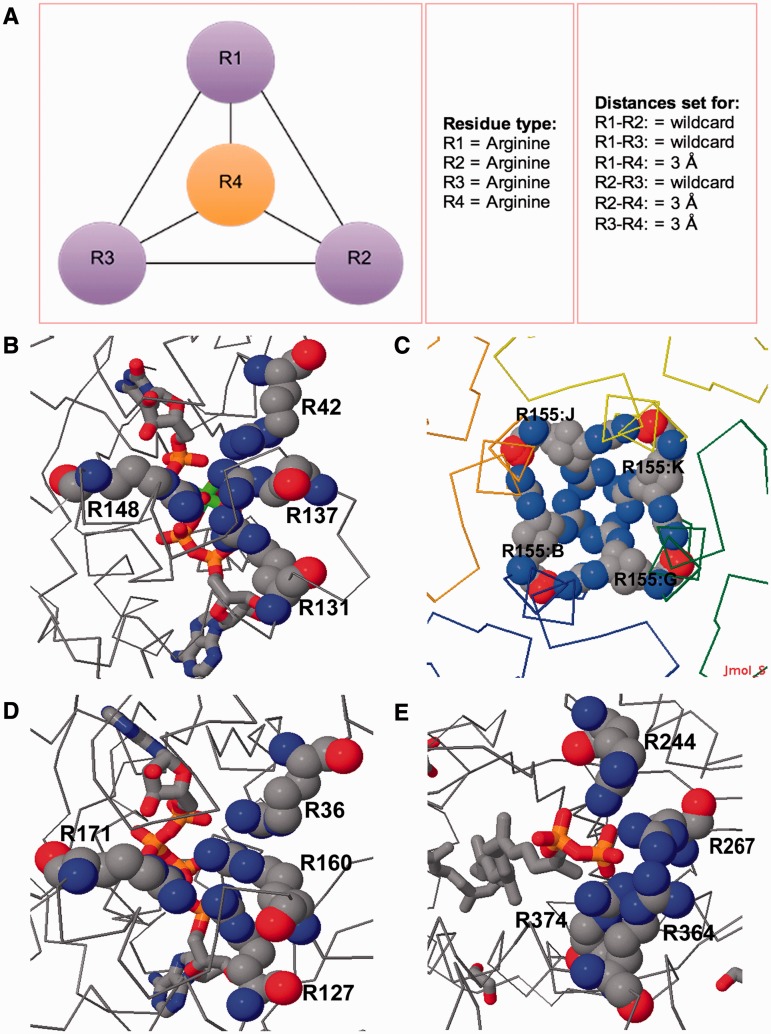
Examples of IMAAAGINE searches for a surrounded residue query. (**A**) The search pattern consists of an arginine surrounded by three other arginines with the distance of each surrounding arginine side chain to the central residue being 3 Å. (**B**) Hit returned for the query in (A) in the structure of slime mold UMP/CMP kinase (PDBID: 3UKD); (**C**) in *E. coli* bacterioferritin (PDBID: 3GHQ); (**D**) in *B. subtilis* adenylate kinase (PDBID: 2QAJ); and (**E**) in *S. pneumoniae* LytR-Cps2a-Psr family protein (PDBID: 3TFL).

The IMAAAGINE query interface offers the user 11 initial options for initiating the design of a query pattern. These options range from a three-residue pattern to an eight-residue pattern in which some or all possible inter-residue distances can, if wished, be defined (Figure 2A). The 4–8 residue searches include an extra option that simplifies the design of a query where one residue or residue type is surrounded by other residues or residue types, and the number of definable distances is reduced (Figure 3). Once the number of residues for a query has been selected, the user can then proceed to design the arrangement pattern for amino acid side chains for searching against the PDB. The current search database is a manually curated non-redundant version of the PDB consisting of 66 575 structures that have been converted into the Key pseudo-atom representations. The search database reported in this article was generated on 6 February 2013 and will be periodically updated with newly available structures on a monthly basis.

The IMAAAGINE interface enables a user to envision a hypothetical arrangement of side chains by specifying either the residue type or a generic residue type for each amino acid side chain in the pattern using dropdown menus (Figure 2A). In addition to the standard amino acids, a number of generic amino acid types are available, facilitated by the use of the single Key atom description. They are as follows: aromatic (Tyr, Phe or Trp); basic (Lys, Arg or His); basic excluding His; acidic (Glu or Asp); amide (Gln or Asn); small hydroxyl (Ser or Thr); medium hydrophobic (Leu, Ile or Val); and hydrophobic (Leu, Ile, Val, Ala, Pro, Met, Phe, Trp or Tyr).

Once the residue or residue types have been specified, the user can then input the distances that define the arrangement. Although the user can input any value into the query field, the interface also allows the possibility of using pre-set distances for interactions such as hydrogen bonds (pre-set at 3 Å), Van der Waals contact (pre-set at 4.5 Å) and disulfide bonds (pre-set at 1 Å) by entering the letters H, V or S, respectively, into the search box. In cases when a search question arises that requires a less tightly defined search, the user can leave the search box empty, and this will result in that particular distance being able to take any value in the retrieved structures. Those distances that are defined have a default search tolerance of 1.5 Å applied to them, which gives flexibility to the search whilst allowing for the positioning of the Key atoms in the side chain. However, users have the option of lowering or increasing this value from the default via the form field provided in the search interface. Lower tolerances can allow a more precise definition of a query, whereas larger ones may be appropriate where larger side chains or longer inter-residue distances are involved.

The principle behind IMAAAGINE is to allow the creation and investigation of ‘broad brush’ queries of the kind ‘give me all circular arrangements of six alternating serine and carboxylic acid residues’ (see Case Study 1 and Figure 2) or ‘show me all instances of an arginine guanidinyl group surrounded by three other guanidinyl groups’ (see Case Study 2 and Figure 3). For such queries, the relative positions of the groups are important, but their precise angular orientations are not. It is therefore advantageous—through the use of appropriate tolerances—to allow for this and also for the fact that some residues may be able to undergo rotation or motion in different structures of the same protein. The time taken to execute an IMAAAGINE search varies and depends on the input specified, the size of the query and the server load at the time the query is submitted. A search carried out on a light server load for a six residue test example took ∼7 min to complete.

## IMAAAGINE: OUTPUT AND VISUALIZATION INTERFACE

Examples of the outputs from IMAAAGINE runs are shown in Figures 2 and 3 and are discussed in more detail in the Case Studies. In essence, there is a summary of the input pattern followed by a list of hits detected in the PDB. A summary of the results is presented to provide information such as the total number of hits that have root mean square deviation (RMSD) values <2 Å and the number of hits where the residues occur only in the same chain. The results presented have been screened to filter out repeat occurrences of the same hits in different sequence order that are present in the raw output as a result of the program’s search approach, which is non-sequential. Because the search is subject to a tolerance (by default of 1.5 Å), hits may match the pattern to a greater or lesser extent, and the goodness of fit is expressed as an RMSD value between the distances defined in the pattern and the equivalent distances found in the matched structure; the output results are hence sorted in ascending order of RMSD so that the earlier hits in the list will be better matches to the query pattern than will the later ones. Each hit is listed by PDB identity code with a link to that entry in the RCSB PDB (1), the name of the protein, the RMSD, the resolution at which the structure was determined, the residues identified as matches, the shortest distance from a hit residue to the nearest non-water hetero atom and what those hetero atoms are; finally, there is a link that allows the user to view the hits in a Jmol (http://www.jmol.org/) viewer window. The structure resolution information is provided owing to the fact that higher resolution structures are likely to be better defined, and this allows users to restrict their analyses to those structures only. Additionally, users can opt to view a list of hits that are entirely within the same chain of the PDB structure. We have demonstrated how both these features are applied during the analysis of IMAAAGINE search results in the ‘Case Studies’ section.

## CASE STUDIES

### A circular serine-carboxyl triplet

As previously mentioned, IMAAAGINE searches can be targeted at partially defined curiosity-driven queries, thus addressing the paucity of computational tools that are able to carry out such searches. To test the capacity of IMAAAGINE to carry out a search for such a hypothetical arrangement, we queried the PDB for a six-residue arrangement that consists of three serine residues alternating with three acidic residues in a circular arrangement where each serine is separated by a 3 Å distance from at least two other acidic residues in the query pattern, and vice versa. All the other distances were left as wildcard values (Figure 2A). Such a query will in effect identify circular arrangements of S-[D/E]-S-[D/E]-S-[D/E] that satisfy the pre-set distances of 3 Å plus or minus the search tolerance. Only distances between potential neighbouring residues are defined, whereas the others have been left blank and may therefore adopt any value. This means that as long as those short range distances are satisfied (within the pre-set tolerance of ±1.5 Å, or by a user-defined tolerance), the actual arrangement retrieved need not be strictly circular but could be elliptical or could be some more complex 3D arrangement of serines and acidic groups. This search returned five hits, of which four were for SDSDSD (Figure 2B), whereas one was found for a mixture of Ds and Es. These results were then visualized using the Jmol plug-in (Figure 2C). The closest hit—although the contacts between the serine OG and the carboxyl oxygens are all >3 Å—is the only example of an SESESD fitting the query and is found clustered on one chain in a sodium/calcium ion exchanger [PDBID: 3V5S (12)] where the three acidic groups and two serine hydroxyls are all in contact with a Cd^2+^ ion. This is one of a cluster of metal-binding sites that appear to be important in the function of this protein (12).

The second hit was found to be an SDSDSD arrangement that is repeated in three different chains of a trimeric phage lyase structure [PDBID: 2X3H, (13)]. These SD pairs appear to be interfacing residues between the three identical subunits (Figure 2D). Owing to this six residue tertiary motif being formed by the trimer assembly, such a motif would most likely be undetectable at the sequence level. The same is true of another trimeric hit in a metal-binding phage baseplate assembly protein [PDBID: 3AQJ (14)] where the aspartates coordinate a Ca^2+^ ion. This example demonstrates that the systematic design of queries, followed by computational screening of IMAAAGINE outputs, has the potential of yielding novel amino acid tertiary motifs that are not detectable via currently available sequence database searching methods.

### Four arginine cluster

Recently, Neves *et al.* (15) published a survey of unusual structures involving stacked arginine guanidinium groups, thus discovering instances of rings of arginines with four to eight members (usually on symmetry axes in oligomeric proteins), stacks of three guanidinium groups, strings of stacked arginines and also ‘planar stacking’ of arginines bridged by hydrogen bonds to other ligands. These patterns are all based on underlying motifs of one arginine stacked against one or two others, as originally identified by Scheraga and colleagues (16). We therefore attempted to extend this analysis by designing a search pattern intended to discover if there were any instances of one arginine guanidinium group surrounded by three others (Figure 3A).

Surprisingly, an IMAAAGINE search found a number of such arginine clusters (Figure 3B–E). The lowest RMSD hit was in the 2 and 1.9 Å resolution structures of uridine/cytidine monophosphate (UMP/CMP) Kinase [PDBID: 2UKD and 3UKD, (17)] where the guanidinyl group of R137 is surrounded by those of R42, R131 and R148 (Figure 3B). R42, R137 and R131 form a stack, and R148 has a planar stack against R137/R131. These residues are in close proximity to the cytidine 5′ monophosphate (C5P) and ADP binding sites with R42 being 3.7 Å from C5P and R131 being 3.8 Å from ADP.

The second hit was in the 2.7 Å structure of *Escherichia coli* bacterioferritin [PDBID: 3GHQ (18)], where four copies of the same arginine (R155) are arranged around a non-crystallographic 4-fold channel through the bacterioferritin shell (Figure 3C). In this case, the arginines are not stacked, but the NH1 of each one is only 2.1 Å from the NH2 of the next. This rather unusual mode of interaction may warrant a confirmatory re-examination of parts of the model derived from this medium resolution refinement.

Therefore, restricting ourselves to hits in structures at resolutions better than 2.0 Å, an IMAAAGINE search hit in the structure of a thermostable mutant of *Bacillus subtilis* adenylate kinase at 1.8 Å (PDBID: 2QAJ, Figure 3D) revealed a cluster of arginines where R36, R160 and R127 form a stack and the fourth arginine (R171) is to one side. This arrangement is similar to that found in the UMP/CMP kinase and is therefore a combination of a three-arginine stack (residues 42, 137, 141 in 2UKD and 36 160 127 in 2QAJ) and a planar stacking of a fourth arginine residue (residue 148 in 2UKD and 171 in 2QAJ) to one in the stack with a linking hydrogen bond from the ligand (Figure 3B and D). Essentially similar hits were also found in several other adenylate kinase structures from other species. A similar arrangement but with the fourth arginine in a plane with the second in the three-arginine stack was found in guanylate kinase (PDBID: 1LVG).

In the 2.1 Å structure of malic enzyme 2 [PDBID: 1QR6, (19)], a different arrangement was found in which four arginines (91:A, 1091:B, 1128:B, 128:A) are clustered together, but none are stacked. Another unstacked arrangement can also be observed in the 2.05 Å structure *of Streptococcus pneumoniae* LytR-Cps2a-Psr family protein [PDBID: 3TFL, (20), Figure 3E]. This unstacked arginine quadruple is found interacting with the diphosphate group of an octaprenyl pyrophosphate lipid. Three arginines (R364 and R374, which are stacked, and R267) make hydrogen bonds to the terminal phosphate where the fourth (R244) bridges between the two phosphates (Figure 3E).

### One protein, two Asp-His-Ser triads?

Because search patterns can contain wildcard (i.e. undefined) distances between residues, it is possible using the IMAAAGINE input interface to devise patterns where one subgroup of residues in the pattern has no defined spatial relationship to the others. This can be valuable in carrying out searches to find two different patterns in the same protein or the simultaneous presence of two similar patterns.

**Figure 4.**
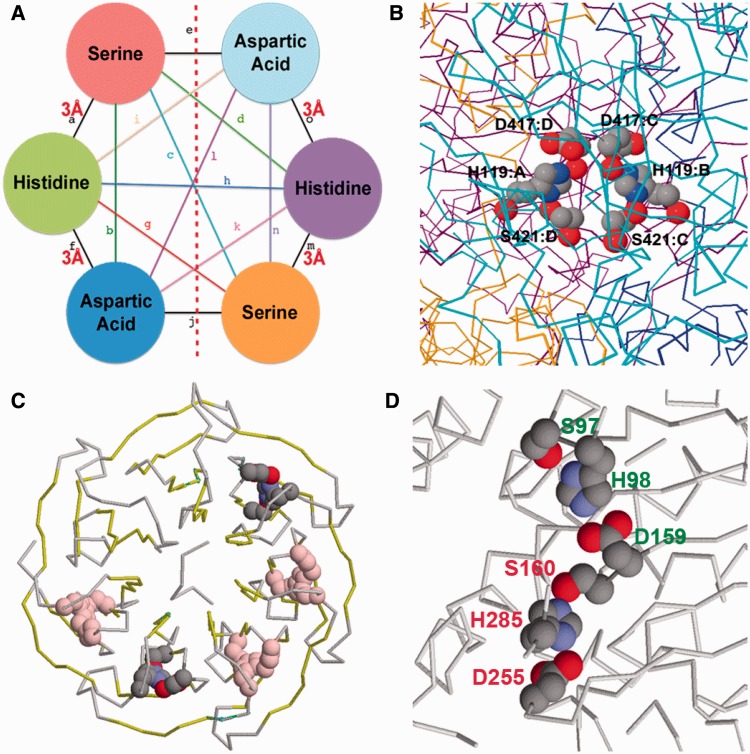
Results from the double Asp-His-Ser triad search. (**A**) The query pattern: no distances are defined between the triad on the left and that on the right, and there are consequently no restraints imposed between the side chains on either side of the red dotted line. (**B**) Two triads related across subunit interfaces in *E. coli* KatE (PDBID: 4ENP). (**C**) One of the pairs of triads found in WDR5 (PDBID: 3EMH) is shown in cpk colours, the other triads being shown in pink, and the beta strand ‘blades’ of the beta propeller shown in yellow (**D**) The pair of triads found in a hyperthermophilic carboxylesterase (PDBID: 1JJI) that involve two consecutive residues D159 and S160 in different triads. The triad labelled in red is the catalytic triad, the other triad is labelled in green.

As an example of this, a search was carried out for structures in the PDB containing two copies of a chymotrypsin-like Asp-His-Ser triad (21). This search motif is shown schematically in Figure 4A. The distances defined are based on the approximate distances in the chymotrypsin catalytic triad (21) allowing for the fact that a tolerance of ±1.5 Å will be applied in the search process. However, no distances are defined between the triad on the left and the one on the right (Figure 4A), and therefore there are no constraints on the relative positions of any pair of triads retrieved.

One hit for the double triad query was in the *E. coli* KatE catalase structure [PDBID: 4ENP, (22)] where the serine and aspartic acid residues of one triad are on one chain (S421:C, D417:C), whereas the histidine is on another chain (H119:B; Figure 4B). This arrangement is repeated for the other triad as well (S421:D, D417:D, H119:A; Figure 4B). This further illustrates the importance of such amino acid arrangements when considered as a tertiary motif that is not an obvious motif candidate when viewed only from a sequence level perspective.

However, as might be anticipated, in a great majority of cases, the pairs of triads retrieved were simply from multiple copies of the same molecule in the asymmetric unit of the crystal. The IMAAAGINE results browser has an option to remove these by screening for hits where all the matches occur only in a single chain. Therefore, to exclude these mostly uninteresting instances, only hits where all six residues were found in the same chain of the protein were examined. A number of hits were found in certain beta propeller structures where many of the repeating blades were found to contain a triad-like motif, thereby occasioning multiple hits. These proteins included the F-box/WD repeat protein 7 [complexed with S-phase kinase-associated protein 1 A, PDBID: 2OVP, (23)], the guanine nucleotide-binding protein subunit beta-like protein (ASC1, RACK1) in the structure of the eukaryotic ribosome [PDBID: 3U5C, (24)] and in other WD40 domains including those of histone-binding protein RBBP4 [PDBID: 3GFC, (25)] and WD40 protein Ciao1 (PDBID: 3FM0).

In the context of a beta propeller and in the absence of a candidate for the oxyanion hole structure that is necessary for stabilizing a catalytic intermediate in serine proteases (21), these triads are unlikely to be catalytic in function, and no catalytic function has yet been observed in a WD40 protein; instead, they act as rigid scaffolds in the molecular recognition of other protein or nucleic acid molecules (25). Nevertheless, by analogy with the serine proteases, the presence of the triad-like motif may create a stronger partial negative change on the serine OG or a stronger positive charge on the imidazole of the histidine, thereby possibly strengthening inter-blade interactions. The highest resolution of the beta propeller structures retrieved in the search is the 1.3 Å structure of WDR5, a component of the mixed lineage leukemia complex [PDBID: 3EMH, (26)], and one of the multiple hits in this protein is shown in Figure 4C. In each case, the serine and the aspartate are on one blade of the propeller, and they are linked by the histidine from a loop connecting the previous blade. The histidine is from a GH dipeptide characteristic of WD40 proteins and is known to participate in a hydrogen bonding network and to strengthen the inter-blade interaction (25). However, although this is a seven-bladed propeller, there are 10 hits indicating that there is a total of only five distinct triads (Figure 4C). As this suggests, two of the seven inter-blade regions lack this interaction: although an aspartate is present at approximately the correct position, the serines and histidines are absent. These differences, together with insertion elements (25), may play a role in bestowing ligand-binding specificity on the surface of this otherwise highly symmetrical structure. The serine in each triad in turn forms a hydrogen bond to a tryptophan in a different strand of the same blade (Figure 4C). Clearly, the tryptophan could also be added to the search pattern if a more specific query were required, and searches could be conducted for either single or double occurrences of this new motif, illustrating how IMAAAGINE results can suggest further searches that can be readily defined and carried out.

Other non-propeller chains contained pairs of triads often separated by long distances. In the hyperthermophilic carboxylesterase from the Archaeon *Archaeoglobus fulgidus* [1JJI, (27)], a number of triads were found, two of which involve the sequence consecutive residues Asp 159 and Ser 160 participating in two different triads to form a linked cluster of two triads (Figure 4D). One triad, D159, H98, S97, has no known function but can be expected to play a role in stabilizing the structure of this hyperthermophilic organism, but the other triad, D255, H285, S160, is the catalytic triad itself (27). This final example illustrates the fact that patterns submitted to the IMAAAGINE server need not be purely conceptual but can be designed on the basis of known patterns from real proteins—in this case, the Asp-His-Ser triad—that can either be simplified, or made more generic, or placed into novel contexts.

### Comparisons with other methods

It is useful to compare the IMAAAGINE service with other 3D protein-searching web servers. A key point here is that most such programs, e.g. ASSAM/SPRITE (8), RASMOT-3D PRO (3), SPASM (6), SA-Mot (28), allow neither searching for motifs with partially defined distances nor angular variation in the relative dispositions of side chains. However, PDBeMotif [formerly known as MSDmotif (29)] is an exception to this. PDBeMotif is a powerful and general relational database-based program that enables the construction of complex and precise queries relating sequence motifs in 3D structures, in addition to much other detailed structural information. These sequence motifs can include single amino acids, and the user can define a subset of distances between them so that it is possible to specify searches in PDBeMotif that parallel those described here for IMAAAGINE. However, direct comparisons proved impossible because PDBeMotif only finds substructures if all the amino acids are in the same chain because its database only stores the pre-computed distance information within each single protein chain (29) in a PDB file. PDBeMotif therefore did not retrieve any of the structures we have described earlier in the text where amino acids come from different subunits, e.g. the serine-carboxyl triplets from three different subunits in 2X3H and 3AQJ, the four-arginine patterns from 3GHQ and 1QR6 or the double triad example from 4ENP. Moreover, the output of PDBeMotif does not distinguish between side chain:side chain, side chain:main chain and main chain:main chain contacts. We also found it would not accept queries where the spatial relationship between two different parts of the pattern is left undefined, as in the double triad example discussed earlier in the text. Therefore, although PDBeMotif is a powerful and general program capable of answering complex queries, IMAAAGINE offers important advantages in the specific application of side chain:side chain searching, and therefore its search facilities constitute a valuable new addition to the existing capabilities for protein side chain motif searching.

## SUMMARY

The IMAAAGINE server allows users to easily propose and then investigate the possible occurrence of novel, hypothetical patterns of amino acid side chains in 3D structures without the need of a known precedent and without requiring the building or creation of a 3D model before the search can be conducted. A simple open-ended searching regime is facilitated by the use of a minimal side chain representation, the use of generic side chain types and the need to specify only a subset of inter-residue distances.

## FUNDING

Ministry of Higher Education Malaysia and Universiti Kebangsaan Malaysia [ERGS/1/2011/STG/UKM/01/15 and UKM-DLP-2012-018]. N.N. acknowledges financial support received from the National Science Fellowship, Ministry of Higher Education, Malaysia. Funding for open access charge: [grant—UKM-DLP-2012-018].

*Conflict of interest statement*. None declared.
